# The Combinational Role of Vitamin D and Other Conditions in Multiple Sclerosis Patients

**DOI:** 10.4314/ejhs.v32i1.26

**Published:** 2022-01

**Authors:** Chrissa Sioka

**Affiliations:** 1 Department of Nuclear Medicine, Medical School, University Hospital of Ioannina, Greece

In a study by Ayele et al, the authors described low vitamin D (Vit D) in 96% of 25 Ethiopian multiple sclerosis (MS) patients, with mean age of 35.8 years (1). Among them, 50% of the patients had severe hypovitaminosis D. Furthermore, lower levels of Vit D were found in older patients and with increased duration of their disease. As the authors state, little is known about MS in Ethiopia (1).

Vitamin D is necessary for bone health and mediates its function via the Vitamin D Receptor (VDR). The VDR gene may exhibit several polymorphisms, some of which have been associated with MS and osteoporosis (2,3). MS patients exhibit increased frequency of low bone mineral density (BMD) which may lead to osteoporosis compared to control individuals, with male patients affected in both lumbar spine and hip, whereas the females are predominantly affected in the hip (4,5). In female patients, however, other factors except low Vit D may influence BMD, such as gynecological factors (6), similarly with healthy females (7,8). Furthermore, MS patients exhibit several mood disorders, with depression seen most often, and also linked to lower Vit D levels (9). A recent study has also described an association between low Vit D levels, depression and osteoporosis with multiple sclerosis (10).

Thus, since the authors stated that it is little known about MS in Ethiopia, I believe that it would be imperative to further study the vitamin D gene polymorphisms, BMD, osteoporosis, and depression, in Ethiopian patients with MS. Future studies should focus on identifying the molecular/genetic factors behind this association in the Ethiopian population.

## References

[1] Ayele BA, Wuhib MZ, Zenebe BG, Metaferia GZ (2021). Serum Vitamin D Level among Multiple Sclerosis Patients in the Tropics: Experience from a Private Clinic in Addis Ababa, Ethiopia. Ethiop J Health Sci.

[2] Sioka C, Kyritsis AP, Fotopoulos A (2009). Multiple sclerosis, osteoporosis, and vitamin D. J Neurol Sci.

[3] Sioka C, Papakonstantinou S, Markoula S, Gkartziou F, Georgiou A, Georgiou I (2011). Vitamin D receptor gene polymorphisms in multiple sclerosis patients in northwest Greece. J Negat Results Biomed.

[4] Sioka C, Fotopoulos A, Georgiou A, Papakonstantinou S, Pelidou SH, Kyritsis AP (2011). Body composition in ambulatory patients with multiple sclerosis. J Clin Densitom.

[5] Sioka C, Papakonstantinou S, Fotopoulos A, Alamanos Y, Georgiou A, Tsouli S (2011). Bone mineral density in ambulatory patients with multiple sclerosis. Neurol Sci.

[6] Sioka C, Fotopoulos A, Papakonstantinou S, Georgiou A, Pelidou SH, Kyritsis AP (2015). The effect of menarche age, parity and lactation on bone mineral density in premenopausal ampulatory multiple sclerosis patients. Mult Scler Relat Disord.

[7] Sioka C, Fotopoulos A, Georgiou A, Xourgia X, Papadopoulos A, Kalef-Ezra JA (2010). Age at menarche, age at menopause and duration of fertility as risk factors for osteoporosis. Climacteric.

[8] Sioka C, Bougias C, Papadopoulos A, Fotopoulos A (2007). Is osteoporosis in postmenopausal female patients related to previous pregnancies and/or miscarriages?. Climacteric.

[9] Raimo S, Santangelo G, Trojano L (2021). The emotional disorders associated with multiple sclerosis. Handb Clin Neurol.

[10] Sioka C, Baldouma A, Papadopoulos A (2021). Co-Existence of Depression, Low Bone Mineral Density, and Vitamin D Deficiency in Patients with Multiple Sclerosis. Psychiatr Danub.

